# How Dietary Phosphorus Availability during Development Influences Condition and Life History Traits of the Cricket, *Acheta domesticas*

**DOI:** 10.1673/031.011.6301

**Published:** 2011-05-17

**Authors:** Laksanavadee Visanuvimol, Susan M. Bertram

**Affiliations:** Department of Biology, Carleton University, I 125 Colonel By Drive, Ottawa, Ontario, Canada KIS 5B6

**Keywords:** *Acheta domesticus*, growth, juvenile, nitrogen, fertilizer, behavioural stoichiometry, nutritional ecology, fitness

## Abstract

Phosphorus is extremely limited in the environment, often being 10–20 times lower in plants than what invertebrate herbivores require. This mismatch between resource availability and resource need can profoundly influence herbivore life history traits and fitness. This study investigated how dietary phosphorus availability influenced invertebrate growth, development time, consumption, condition, and lifespan using juvenile European house crickets, *Acheta domesticus* L. (Orthoptera: Gryllidae). Crickets reared on high phosphorus diets ate more food, gained more weight, were in better condition at maturity, and contained more phosphorus, nitrogen, and carbon in their bodies at death than crickets reared on low phosphorus diets. There was also a trend for crickets reared on high phosphorus diets to become larger adults (interaction with weight prior to the start of the experiment). These findings can be added to the small but growing number of studies that reveal the importance of phosphorus to insect life history traits. Future research should explore the importance of dietary phosphorus availability relative to protein, lipid, and carbohydrate availability.

## Introduction

Organisms require nitrogen and phosphorus to build their proteins, RNA, DNA, and ATP (Sterner and Elser 2002). Obtaining enough of these limiting resources can be difficult, however, as they must be acquired through the diet and they are often extremely limited in the environment (White 1993). Nitrogen and phosphorus content tends to be 10–20 times lower in plants than in herbivores; this stoichiometric mismatch can hinder the ability of herbivores to meet their nutritional demands (Mattson 1980; Elser et al. 2000a). Not meeting nutritional demands has the potential to constrain organismal life-history traits and fitness (Mattson 1980; Strong et al. 1984; Elser et al. 2000a).

For decades, a mismatch in nitrogen content between insects and their food plants has been recognized to be a critical factor influencing herbivore success (Slansky and Feeny 1977; McNeill and Southwood 1978; Mattson 1980; Denno and McClure 1983; Strong et al. 1984; White 1993; Schindler and Eby 1997). Aphids, chinch bugs, lepidopterous larvae and mites, for example, show increased growth rates, longevity and survival when they consume foods with elevated nitrogen content (Rodriguez 1960; Cannon and Connell 1965; Storms 1969; Soo Hoo and Fraenkel 1966; Van Emden 1966; Dixon 1970).

Although nitrogen has traditionally been considered *the* essential limiting element, phosphorus is also limited in a diverse number of ecosystems (Redfield 1958; Schindler 1977; Hecky and Kilham 1988; Karl et al. 1995; Tanner et al. 1998). An uneven distribution in phosphorus content between herbivores and their diet is also thought to be a critical factor influencing herbivore success (Fox and Macauley 1977; Sterner and Schulz 1998). For example, *Daphnia obtusa* fed phosphorus insufficient algae grow at slower rates and produced smaller eggs compared to those fed phosphorus sufficient algae (Sterner et al. 1993; Schulz and Sterner 1999; Urabe and Sterner 2001). Further, experimental studies have revealed that phosphorus is a growth-limiting element for many organisms including bacteria (Vadstein 2000), algae (Rothhaupt 1992), and Zooplankton (DeMott et al. 1998; Elser et al. 2001). While most research on dietary phosphorus has focused on aquatic invertebrates, reduced availability of phosphorus in the diet has been shown to decrease growth rates in caterpillars and plant hoppers (Perkins 2004, Huberty and Denno, 2006).

Because of its tight link with invertebrate growth (Sterner and Hessen 1994; Quraishi et al. 1966; Elser et al. 2000a; Elser et al. 2001; Urabe and Sterner 2001; Eskelinen 2002; Elser et al. 2003; Schade et al. 2003), phosphorus availability during development may be an important factor influencing life history traits. This link between phosphorus availability and invertebrate growth has been hypothesized to be driven by cellular allocation to phosphorus rich ribosomal RNA (rRNA) (the Growth Rate Hypothesis; Elser et al. 2000b). rRNA is important to growth as it makes up 50–60% of the ribosome, the growth machinery of the cell (Becker 1986), and several million ribosomes are necessary to support protein synthesis in an average cell (Lewin 1980). At steady state, rRNA usually comprises 80–90% of the total cellular RNA, with increasing total allocation at high growth rate (Alberts et al. 1983). Because RNA is almost 10% phosphorus by weight, differences in the availability of phosphorus in the diet may explain the link between phosphorus availability and growth rate (Sterner et al. 1995; Elser et al. 1996; Elser et al. 2000a). The phosphorus rich signature of rapid growth (Main et al. 1997; Watts et al. 2006) may therefore be a cellular necessity, derived from the fact that ribosomes are unusually rich in phosphorus (Elser et al. 1996). A consistent positive association should therefore be expected between growth rate, RNA concentration, and percent phosphorus.

There is substantial support for the growth rate hypothesis. Growth rate, RNA concentration, and percent phosphorus are strongly associated: positive correlations have been found between phosphorus and growth rate (Sutcliffe 1970) and between RNA content and growth rate. Recent experimental studies of phosphorus limitation in insects have also revealed a growth rate affect. For example, caterpillars (*Manduca sexta*) reared on natural and artificial diets with reduced levels of phosphorus exhibited significantly decreased growth rate and increased time to final moult compared to caterpillars reared on phosphorus sufficient diets (Perkins et al. 2004). Laboratory-reared juvenile mayflies fed low phosphorus diets also had significantly reduced growth rates compared to mayflies fed high phosphorus diets (Frost and Elser 2002). Further, RNA concentrations and total body phosphorus content were positively correlated with ontogenetically based changes in growth rate in the fruit fly, *Drosophila melanogaster* (Watts et al. 2006). Additionally, the fraction of total body phosphorus that contributed to rRNA increased with increasing growth rate (Watts et al. 2006). Because growth rates are central to life history theory, the growth rate hypothesis links the availability of essential but limiting elemental nutrients to evolutionary questions.

The time has come to explore the affects of varying phosphorus diets on insect life history traits. European house crickets, *Acheta domesticus* L. (Orthoptera: Gryllidae), were used as a model organism. Male cricket lifetime reproductive success is dependent upon their ability to attract mates. Cricket mate attraction abilities are, in turn, dependent upon acoustic mate attraction signal quality and quantity (Alexander 1961; Hedrick 1986; Zuk 1987; Cade and Cade 1992; Wagner et al. 1995; Gray 1997; Nelson and Nolen 1997; Hedrick and Weber 1998; Gray and Cade 1999; Wiegmann 1999). Previous research on adult male *A. domesticus* revealed that signalling effort was positively influenced by dietary phosphorus availability (Bertram et al. 2009). Given body size and condition also influence signal quality (Gray 1997), it is important to quantify how dietary phosphorus availability during development influences juvenile cricket growth, adult body size and adult condition. Female cricket lifetime reproductive success is dependent upon the number and quality of eggs laid. Previous research on adult female *A. domesitcus* revealed that reduced phosphorus diets lowered the propensity of laying eggs. Further, females on reduced phosphorus diets laid significantly fewer eggs than females on high phosphorus diets (Visanuvimol and Bertram 2010). Given body size and condition also influenced egg numbers (Visanuvimol and Bertram 2010), it is important to understand how dietary phosphorus availability during development influences juvenile growth, adult body size and adult condition. The purpose of this study, therefore, is to quantify how the availability of dietary phosphorus during development affects cricket weight gain, body size and condition at adulthood, and lifespan.

## Materials and Methods

European house crickets, *A. domesticus*, were purchased as third and fourth instar juveniles from Port Credit Pet Center in Port Credit, Ontario, Canada. They were raised communally in 36-litre rectangular plastic containers (36 L × 28 W × 23 H cm) in an insect rearing facility in the Nesbitt Biology Building at Carleton University. The rearing facility had a temperature range of 26 ± 4°C and 12 hour light :12 hour dark cycle. All crickets were provided with cardboard cartons for shelter, and unlimited access to food and water. Food consisted of powdered Harlan Teklad Rodent diet no. 8604 (1% phosphorus; manufactured by Harlan Laboratories Inc, www.harlan.com) until the day they were included in the experiment.

The experiment was initiated at the start of the 7^th^ instar to ensure crickets would have two full instars of growth on the phosphorus diets prior to undergoing final moult (*A. domesticus* typically go through eight different instars before moulting into adults; Roe *et al*., 1985). Crickets were examined daily to see if they had reached their 7th instar. The 7th instar is easily recognizable as it is when the wing pads and ovipositior become visible (Roe *et al*., 1985). The day crickets reached their 7^th^ instar they were removed from the rearing colony and housed alone in 500 ml plasticcoated paper bowls (7 H × 11 D cm) with shelter and unlimited access to water.

Approximately 58 males (range = 57–60) and 58 females (range = 53–62) were placed on each of the five different phosphorus diets ranging from low to high phosphorus content (0.2%, 0.4%, 0.6%, 0.8%, 1.0%). This phosphorus range was designed to mimic the range of food in the wild. Since crickets are omnivores they are capable of consuming a variety of foods, from plants to fungi to insects. These foods vary from an average of 0.2% phosphorus found in terrestrial plant matter to an average of 0.8% phosphorus found in insects (Sterner and Elser 2002). Experimental diets were designed, manufactured and purchased from Harlan Laboratories Inc. Most of the phosphorus was delivered using calcium phosphate (1.0%P = 35.53 g/kg; 0.8%P = 26.75 g/kg; 0.6%P = 17.98 g/kg; 0.4%P = 9.21 g/kg; 0.2%P = 0.44 g/kg), however, 1.9 g/kg of phosphorus came from casein which was used as a protein source. Calcium levels were balanced across the diets to 1% with calcium carbonate (1.0%P = 0.0 g/kg; 0.8%P = 6.5 g/kg; 0.6%P = 12.75 g/kg; 0.4%P = 19.25 g/kg; 0.2%P = 25.75 g/kg). Each diet also contained 24% protein (236.6 g/kg of protein; 272 g/kg casein), fat (42.7 g/kg: 2.7 g/kg from casein and 40 g/kg from soybean oil), L-cystine (4 g/kg), corn starch (150 g/kg), maltodextrin (50 g/kg), cellulose (50 g/kg), minerals (13.4 g/kg; note no calcium or phosphorus included in the mineral mix), vitamins (10 g/kg), choline bitartrate (2.5 g/kg) and antioxidants (8.0 mg/kg). Thus, diets were identical in every aspect except for the amount of phosphorus they contained. Crickets were provided with unlimited access to their assigned experimental diet from the day they reached their 7th instar until the day they died a natural death.

### Life history traits

Each cricket's initial body mass was obtained using a Denver Instruments Precision Analytical Balance model P-114 (www.denverinstruments.com) on the day it reached its seventh instar. Change in body mass over time was quantified by weighing each cricket every week until they died naturally.

Crickets were checked daily throughout the experiment to determine if they had moulted to adulthood, escaped from their containers (happened rarely), or died. Developmental time was calculated as the difference between start date (day the individual reached its seventh instar) and the date it moulted to adulthood (adult date). Lifespan was calculated as the difference between the start date and the date each individual died a natural death (death date). Adult lifespan was also quantified using the difference between the date each individual moulted to adulthood and their death date. The few individuals that escaped prior to dying or did not have their death date or maturity date recorded were excluded from analyses.

Each cricket's head width, thorax width, thorax height, and thorax area were measured to the nearest 0.1 mm using a Zeiss Discover 4. V 12 dissection microscope (www.zeiss.com) following their natural death. These size measurements were significantly positively correlated with each other (correlations ranged from 0.6602 to 0.9067; all p-value <.0001). A principal component analysis was used to reduce the number of variables. The 1^st^ principle component explained 98% of the variation in body size (eigenvalue = 15.3259; loadings: thorax width = 0.15, thorax height = 0.10, thorax area = 0.98, and head width = 0.11) and was used as an overall size measure.

**Table 1.**
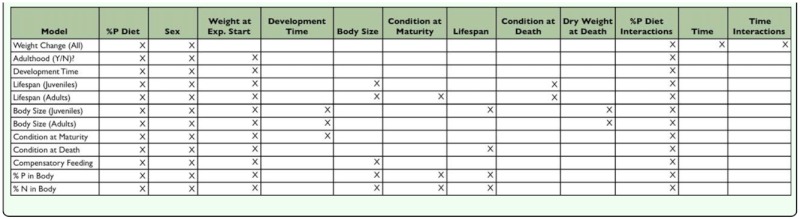
Parameters included in the models used to analyze the factors contributing to life-history traits. Factors (columns) with an × were included in the model (rows).

Cricket condition at maturity was quantified using the residuals from an allometric regression of body weight (wet weight at maturity) on body size. Cricket condition at death was quantified using the residuals from an allometric regression of body weight at death (dry weight) on body size (PC1). To obtain dry weight measures each cricket was dried at 130°C for at least 24 hours in a Thermal Scientific (6520 series) drying oven (www.thermalscientific.com). A temperature of 130° C was used because this was the lowest temperature our drying oven could reach. Unfortunately, this high temperature might have resulted in some of the volatile organics evaporating. If so, the dry weights might be slightly lower than normal. Dry weight was quantified using a Denver Instruments Precision Analytical Balance (model P-114).

### Compensatory feeding

Because some invertebrates compensate for poor quality diets by eating more food (Darchambeau 2005; Hubertry and Denno 2006; Boersma and Elser 2006) it was important to quantify the amount of diet consumed. Cricket consumption rates were monitored daily over a three day period. A subset of juveniles from each of the five diets (at least 10 males and 10 females per diet) were given a pre-weighed amount of food (more food than they could consume in a 24 hr period) on day ten of the experiment. Twenty-four hours later any cricket frass was carefully removed from the food dish and the remaining food was re-weighed. Total food consumption was calculated by determining the difference between the start weight and the end weight (24 hours later) of the food. This process was repeated every 24 hours over the course of three days to obtain three consumption measures per individual. Each individual's average consumption rate was then calculated. Only juveniles that did not moult in the day prior, during, or the day immediately following the compensatory feeding assays were included.

**Table 2.**
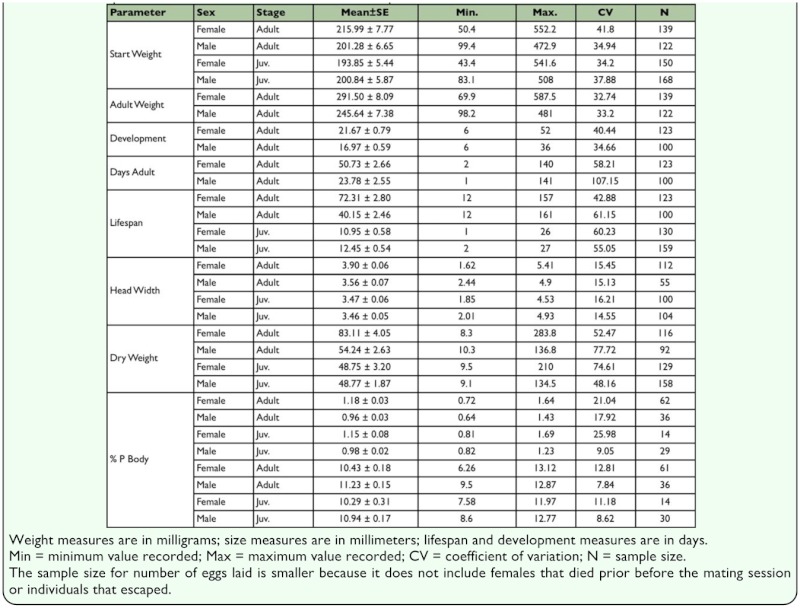
Descriptive statistics for all measured variables in experiment.

### Stoichiometry

Cricket total body phosphorus and nitrogen content were quantified on a subset of the individuals (at least 10 males and 10 females per diet) to ascertain how phosphorus influenced body stoichiometry. Each dried cricket was pulverized to a uniform powder using a mortar and pestle. Approximately 1–2 mg of powder was then used to assess individual body stoichiometry. To quantify total body phosphorus content a persulfate oxidation technique followed by orthophosphate analysis using the acid molybdate technique was utilized (APHA 1992). To quantify total body nitrogen content Elementar (www.elementar.de) Vario Micro Cube CHN analyzer was utilized for the Dumas method. The Dumas method involves combusting the samples at high temperatures using oxygen. The gas that is produced is then passed through a special column where carbon dioxide and water is absorbed. The carbon and nitrogen content is then analyzed from this gas. The product that remains from the combusted sample can then be analyzed for nitrogen content.

### Statistical analyses

Statistical analyses were conducted using JMP 8.0.1 statistical software (SAS Institute Inc. www.sas.com). Shapiro-Wilk goodness-of-fit tests were used to ensure the data do not differ significantly from normality. When data were not normally distributed (lifespan), they were transformed using a log transformation to approximate a normal distribution. A repeated measures analysis of variance was used to determine how body weight changed through time. A nominal logistic fit model was used to explore the factors influencing whether or not crickets survived through adulthood. Multiple regression models were used to quantify whether phosphorus availability influenced compensatory feeding behaviour, body size, condition, and body stoichiometry. Cox proportional hazard survival models were used to determine the factors influencing cricket lifespan (overall), juvenile lifespan (individuals that died as juveniles), and for the individuals that died as adults, juvenile development time and adult lifespan. Parameters and interactions included in each of the multivariate models are shown in Table 1. Correlation analysis was used to examine the relationship between cricket body nitrogen and phosphorus content.

## Results

**Figure 1.**
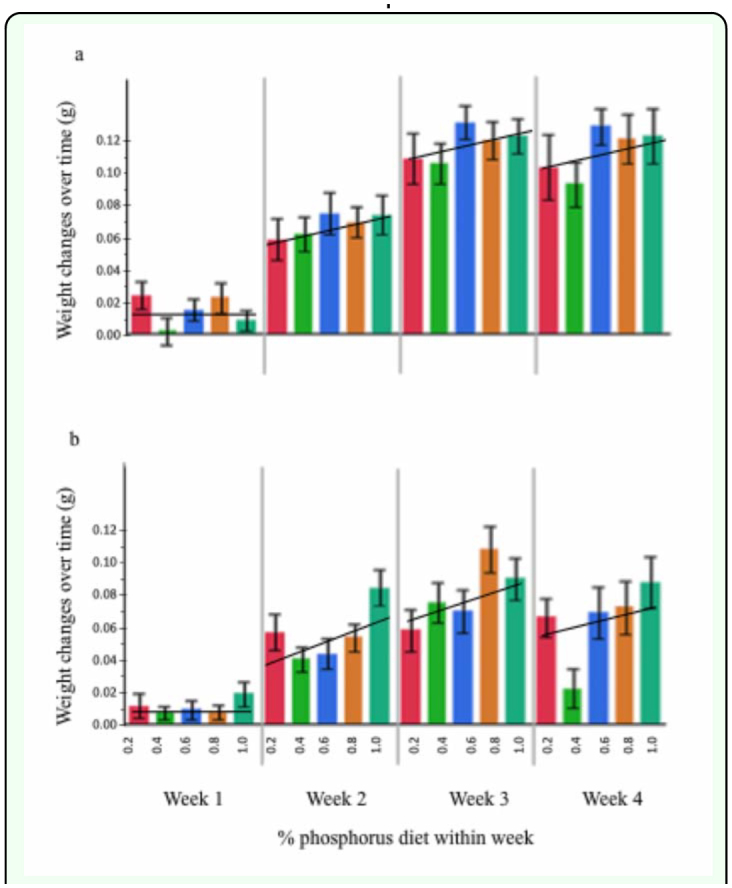
Relationship between the amount of phosphorus in the diet and change in body mass over time for (a) female and (b) male European house crickets, *Acheta domesticus*. Cricket body mass increased over time; changes in body mass were also positively influenced by dietary phosphorus availability. High quality figures are available online.

Cricket weight gain was influenced by the availability of phosphorus in the diet. Overall, juveniles fed enhanced phosphorus diets gained significantly more weight over time than juveniles fed reduced phosphorus diets. Cricket weight gain was also influenced by development time and the sex of the cricket (Figure 1, Table 2,3).

**Table 3.**
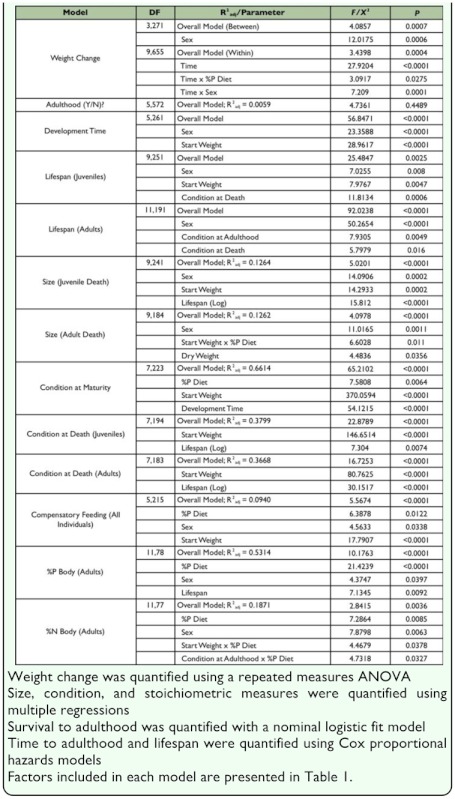
Factors influencing variation in life history traits (factors with P<0.10 included).

Developmental time (time to adulthood/maturity) was not influenced by the availability of phosphorus in the diet. Development time was, however, influenced by the sex of the cricket, with females taking almost five days longer to develop to adulthood than males (Table 2,3). Developmental time was also influenced cricket weight prior to the start of the experiment, with heavier juveniles maturing into adults faster than lighter juveniles (Table 3).

The model exploring survival to adulthood (successful maturation) was not statistically significant (Table 3). Successful maturation was not influenced by the availability of phosphorus in the diet, cricket sex, weight prior to the start of the experiment, or the interaction between these variables and phosphorus availability in the diet.

Cricket lifespan was not influenced by the availability of phosphorus in the diet. Lifespan was, however, influenced by cricket sex, condition at adulthood, and condition at death (Table 3). Females lived longer than males. Crickets that were heavier at the start of the experiment lived longer than lighter crickets.

**Figure 2.**
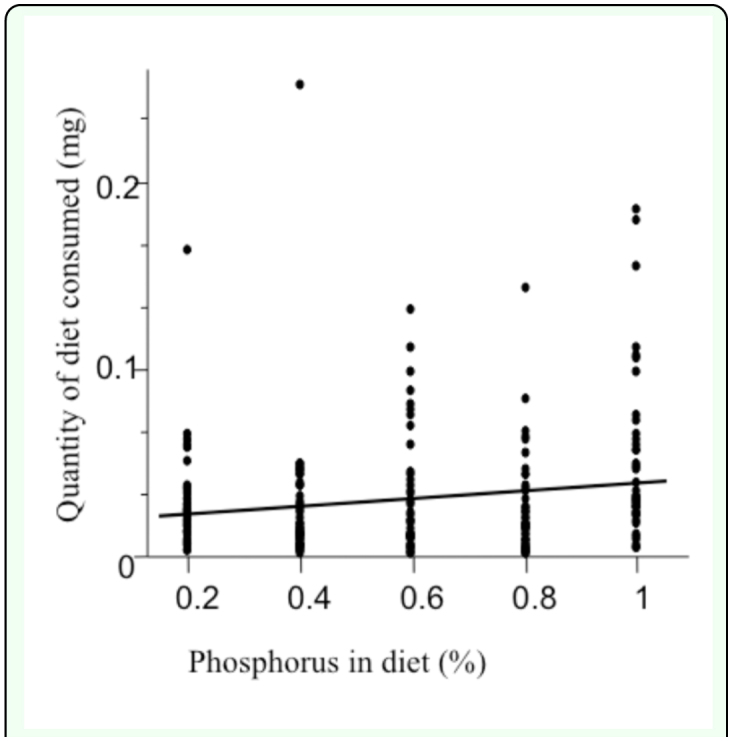
Relationship between the amount of phosphorus in the diet and consumption rate for European house crickets, *Acheta domesticus*. Crickets reared on low phosphorus diets consumed less food per day than crickets reared on high phosphorus diets. High quality figures are available online.

Further, crickets in good condition at adulthood lived longer than crickets in poor condition at adulthood. Crickets that survived a long time also tended to be in better condition at death than crickets that died earlier in the experiment (Table 3).

The factors that influenced adult body size were sex (females were bigger than males) and an interaction between start weight and dietary phosphorus availability (lighter crickets at the start of the experiment were positively affected by phosphorus availability; heavier crickets were not). Together these factors explained 13% of the variation in body size and suggest phosphorus availability positively influences cricket body size, depending on start weight.

Cricket condition at maturity was significantly influenced by the availability of phosphorus in the diet. Crickets with access to high phosphorus diets were in significantly better condition at maturity than crickets reared on low phosphorus diets. Condition at maturity was also influenced by cricket weight prior to the start of the experiment, and developmental time (Table 3). Crickets that where heavy prior to the start of the experiment were also in better condition at maturity. Further, crickets that took longer to develop to adulthood were in better condition at maturity than crickets that developed quickly. Together these factors explained 66% of the variation in condition at maturity.

Cricket condition at death was not influenced by the availability of phosphorus in the diet. It was, however, dependent on cricket weight at the start of the experiment and cricket lifespan (Table 3). Crickets that were heavier at the start of the experiment were in better condition at death. Similarly, crickets that lived a long time were in better condition at death than crickets that died young. Just over one third of the variation in condition at death was explained by these factors.

### Compensatory feeding

Crickets did not compensate for poor phosphorus availability by consuming more food as would be expected if crickets were compensating. Instead, crickets tended to eat less when the diet contained little phosphorus (Figure 2). Foraging behaviour was influenced by start weight and sex. Heavier individuals consumed more food per day than lighter individuals (Table 3). Together these factors explained 9% of the variation in juvenile cricket compensatory feeding behaviour (Table 3).

### Stoichiometry

**Figure 3.**
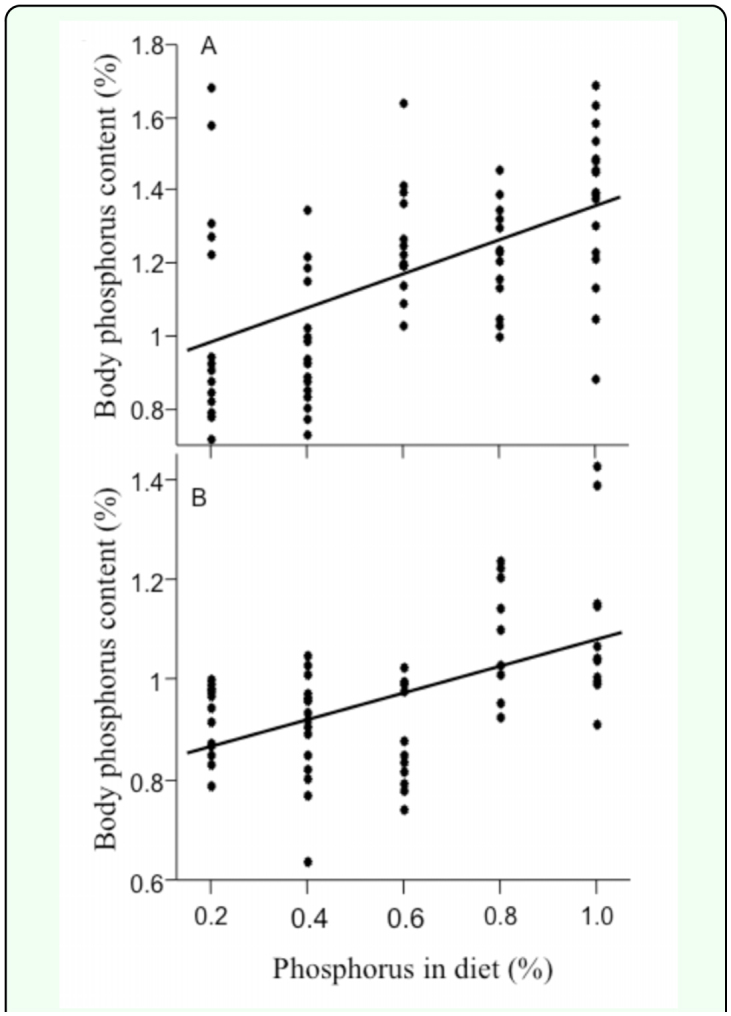
Relationship between the amount of phosphorus in the diet and cricket total body phosphorus content for (a) female and (b) male European house crickets, *Acheta domesticus*. There was a significant positive relationship between phosphorus availability and body content. High quality figures are available online.

Cricket body stoichiometry was highly variable across individuals (Table 2), and the availability of phosphorus in the diet was one of the key factors influencing body stoichiometry (Table 3). Crickets with more phosphorus in their diets had higher body phosphorus contents compared to crickets that with reduced phosphorus diets (Figure 3). Further, cricket sex, lifespan and an interaction between sex and phosphorus availability also influenced cricket body stoichiometry (Table 3; Figure 3). Females had higher phosphorus levels in their bodies than males (Table 2), and female body phosphorus content was more affected by the availability of phosphorus in the diet than male body phosphorus content. Further, crickets that lived longer tended to have more phosphorus in their bodies than crickets that died young. The model for body phosphorus content was statistically significant and explained 53% of the variation in total body phosphorus content.

Cricket total body phosphorus and nitrogen contents were positively correlated with each other (r=0.2829, P = 0.0050, N = 97). Further, nitrogen content was positively influenced the availability of phosphorus in the diet. Nitrogen content was also influenced by cricket sex (males contain more nitrogen than females), an interaction between start weight and phosphorus availability, and an interaction between condition at maturity and phosphorus availability (Table 3). Together these factors explained 19% of the variation in cricket body nitrogen content.

## Discussion

Crickets reared on high phosphorus diets gained weight faster, were in better condition at maturity, and had more phosphorus and nitrogen in their bodies than crickets reared on low phosphorus diets (Table 1,3). High phosphorus diets were also implicated in adult body size (interaction with weight at start of the experiment; Table 1,3). Together, these results suggest that the ability to locate, obtain, and retain dietary phosphorus positively influences cricket life history traits.

The finding that dietary phosphorus availability impacts cricket weight gain (Figure 1) is consistent with other insect studies. Tobacco hornworm larvae (Perkins 2004), benthic mayfly nymphs (Frost and Elser 2002), and southern house mosquitoes (Peck and Walton 2005) reared on phosphorus rich food exhibited elevated growth rates compared to individuals reared on reduced phosphorus diets. Phosphorus is also known to influence growth in a variety of other organisms including bacteria, algae, and Zooplankton (Rothhaupt 1992; DeMott et al. 1998; Vadstein 2000; Elser et al. 2001). Together these findings suggest that phosphorus availability influences growth in most invertebrates and provide support for the growth rate hypothesis.

Increased dietary phosphorus availability did not influence the likelihood that crickets would successfully develop into adults, nor did it shorten the time it took them to undergo their final moult. These findings conflict with those of Perkins et al. (2004) who revealed that increased phosphorus availability shortened the time to final instar moult. These studies are not directly comparable, however, as Perkins et al. (2004) reared tobacco horn worm larvae phosphorus diets throughout juvenile development whereas we reared crickets on phosphorus diets through only the last two of eight juvenile stages (and into adulthood). Future research should examine the effect of reduced phosphorus throughout cricket development.

Dietary phosphorus availability did not directly influence cricket lifespan. This finding supports all other cricket studies to date that have investigated whether phosphorus availability influences length of life (Bertram et al. 2009; Visanuvimol and Bertram, 2010).

Crickets reared on high phosphorus diets were in better condition at maturity than those reared on low phosphorus diets. Further, crickets reared on high phosphorus diets tended to be larger than crickets reared on low phosphorus diets (interaction with body weight). These findings suggest important fitness ramifications, as body size and condition can influence lifetime reproductive success. The quality and quantity of acoustic mate attraction signals are partially dependent on cricket body size and condition (Bertram, unpublished data; Gray 1997). Similarly, female cricket egg laying behaviour is also dependent on cricket body size and condition (Visanuvimol and Bertram 2010). Thus, these results indicate that the availability of dietary phosphorus during adulthood may directly influence both condition and fitness (present study, Bertram et al. 2009, Visanuvimol and Bertram 2010).

### Compensatory feeding

Crickets did not compensate for low phosphorus availability by consuming more food. Instead, crickets reared on high phosphorus diets tended to consume more food than those reared on the low phosphorus diets. In order for crickets to compensate for missing nutrition, they must be able to assess the current state of their food and their own internal elemental component. To date, there has been no research to address whether *A. domesticus* can assess its own internal elemental composition. In caterpillars, the measure of internal state is influenced by the concentration of trehalose, the main storage of sugar in insects, in the haemolymph (Thompson 2003). Haemolymph trehalose concentration increases when caterpillars eat carbohydrate rich foods and decreases when they eat protein rich foods. The process in gustatory response to nutrient stimuli causes the insect to switch between food types (Schiff et al. 1989; Friedman et al. 1991).

The fact that crickets did not compensate for low phosphorus availability by consuming more food does not preclude the possibility that crickets might compensate for low phosphorus availability in other ways. Crickets could potentially select different foods or disperse to new environments with higher quality food when faced with low phosphorus availability. These hypotheses have not been tested as crickets that were used in this experiment were confined to a single food source. Future studies should therefore examine whether crickets preferentially forage on high quality foods or disperse to new environment when given the opportunity.

### Bacterial endosymbionts

Bacterial endosymbionts can play an important role in the metabolism and food budgets of their insect hosts (Douglas 1994; Houk and Griffiths 1980). Insects that possess bacterial symbionts tend to live on nutritionally poor or unbalanced diets during at least one stage in their lives (Douglas 1998). These symbiotic microorganisms are thought to provide a supplemental source of essential nutrients ((Douglas 1994; Houk and Griffiths 1980). Given a wide variety of insects host bacterial symbionts (e.g., Blattaria, Heteroptera, Homoptera, Anoplura, Mallaphaga, Diptera, Coleoptera, and Formicidae; Douglas 1998) these bacterial endosymbionts may also play an important role in cricket metabolism. Unfortunately, to our knowledge, little is known about bacterial endosymbionts in crickets and the role they may play in compensating for reduced phosphorus diets. This may be a fruitful avenue for future work.

### Stoichiometry

Crickets that were fed high phosphorus diets had greater amounts of phosphorus and nitrogen in their bodies than those that were fed reduced phosphorus diets (Figure 4). This begs the question of where the phosphorus and nitrogen reside once the crickets consume it. Phosphorus could be incorporated into cricket RNA (Elser et al. 2000); this is a likely scenario given growth requires extensive protein synthesis and crickets reared on the high phosphorus diets gained more weight over time than crickets on low phosphorus diets. Crickets could also store excess phosphorus in their hemolymph (Woods et al. 2002). Nitrogen is likely incorporated into cricket muscle. To date, none of these hypotheses have been tested in crickets.

### Comparing phosphorus' impact: Juvenile versus adult exposure

The present study was a sequel to the earlier papers that investigated how phosphorus availability during adulthood influenced reproduction (Bertram et al. 2009; Visanuvimol and Bertram 2010). The adult experiments revealed that dietary phosphorus is an important factor influencing reproduction: males with better access to phosphorus signalled with higher effort; females laid more eggs. Both the present study and Visanuvimol and Bertram's (2010) study revealed that crickets do not compensate for poor phosphorus diets by consuming more food. Crickets in the adult experiment did not differ in their foraging behaviour. Although crickets in the juvenile experiment did differ, it was in the opposite direction than predicted: juveniles that fed on reduced phosphorus diets consumed less food, not more. These results suggest that juveniles may be more selective consumers than adults and/or that phosphorus may be more important during development than it is in adulthood.

The juvenile and adult experiments differed in how dietary phosphorus availability influenced body stoichiometry. There was no relationship between dietary phosphorus availability and body stoichiometry in the adult experiment (Visanuvimol and Bertram 2010). However, the present experiment revealed that crickets fed high phosphorus diets contain more phosphorus and nitrogen in their bodies. These differences suggest that the ability to uptake and retain phosphorus has a stronger impact on body stoichiometry during development than it does in adulthood. Other studies also suggest that juvenile body stoichiometry may be influence by dietary intake. For example, *M. sexta* caterpillars fed high phosphorus diets contained significantly more phosphorus it their bodies compared to caterpillars fed low phosphorus diets (Perkins et al. 2004). Together these results suggest that phosphorus availability during development may substantially influence insect body stoichiometry during adulthood.

Together the cricket experiments suggest that cricket growth, condition at maturity, lifetime reproductive efforts, and body stoichiometry are positively influenced by the availability of phosphorus during development and into adulthood (present study, Bertram et al. 2009, Visanuvimol and Bertram 2010). These studies also suggest that neither lifespan nor condition at death are directly affected by the availability of dietary phosphorus.

### Conclusions and future directions

The findings presented herein and in previous published studies support the small but growing number of studies that reveal phosphorus' importance in influencing insect life history traits. Given the plethora of research suggesting that proteins, lipids, and carbohydrates also influence insect life history traits (e.g., Hunt et al. 2004) the importance of phosphorus should be studied relative to these more intensely studied dietary nutrients. In a recent study examining the importance of nutrient availability to grasshopper life history traits, Loaiza et al. (2008) revealed that the need for phosphorus was heavily overshadowed by the need for protein and carbohydrates. A more inclusive investigation of how nutritional ecology influences insect life-history traits and fitness is therefore required (Simpson and Raubenheimer 2000, 2001). Given the plethora of possible essential nutrient and elemental combinations available to choose from (Clancy and King 1993), determining which combinations of nutrients and elements influence insect life history traits fitness remains a major challenge of nutritional ecology.
